# Postmortem Interval Influences **α**-Synuclein Expression in Parkinson Disease Brain

**DOI:** 10.1155/2012/614212

**Published:** 2012-03-13

**Authors:** Alexandra Dumitriu, Carlee Moser, Tiffany C. Hadzi, Sally L. Williamson, Christopher D. Pacheco, Audrey E. Hendricks, Jeanne C. Latourelle, Jemma B. Wilk, Anita L. DeStefano, Richard H. Myers

**Affiliations:** ^1^Department of Neurology, Boston University School of Medicine, Boston, MA 02119, USA; ^2^The Graduate Program in Bioinformatics, Boston University, Boston, MA 02215, USA; ^3^Department of Biostatistics, Boston University School of Public Health, Boston, MA 02118, USA; ^4^Whitehead Institute for Biomedical Research, Cambridge, MA, USA

## Abstract

Duplications and triplications of the *α*-synuclein (*SNCA*) gene increase risk for PD, suggesting increased expression levels of the gene to be associated with increased PD risk. However, past *SNCA* expression studies in brain tissue report inconsistent results. We examined expression of the full-length *SNCA* transcript (140 amino acid protein isoform), as well as total *SNCA* mRNA levels in 165 frontal cortex samples (101 PD, 64 control) using quantitative real-time polymerase chain reaction. Additionally, we evaluated the relationship of eight SNPs in both 5′ and 3′ regions of *SNCA* with the gene expression levels. The association between postmortem interval (PMI) and *SNCA* expression was different for PD and control samples: *SNCA* expression decreased with increasing PMI in cases, while staying relatively constant in controls. For short PMI, *SNCA* expression was increased in PD relative to control samples, whereas for long PMI, *SNCA* expression in PD was decreased relative to control samples.

## 1. Introduction

One of the genes ubiquitously involved in Parkinson disease (PD, OMIM no. 168600) is the **α*-*synuclein (*SNCA*) or *PARK1/4* gene. The *SNCA *gene encodes two major transcripts: the full-length NM_000345.2 transcript and the NM_007308.1 transcript (corresponding to the NACP140, and the NACP112 protein isoforms, resp.). Missense mutations in *SNCA* [1–3], as well as duplications and triplications of the *SNCA* locus [4–6], have been shown to lead to familial PD in an autosomal dominant manner, suggesting that increased levels of *SNCA* are associated with PD risk. Several studies have compared sporadic PD and control *SNCA* mRNA levels, as well as *α*-synuclein protein levels in various tissues [7–14]. *SNCA *expression in human brain has been shown to be significantly different between sporadic PD cases and controls, although the direction of results varies among different studies (Table 1). 

Variations in both the 3′ and the 5′ regions of the *SNCA *gene have also been associated with increased risk for idiopathic PD [15–22]. Additionally, there is evidence that single-nucleotide polymorphisms in the 3′ region of *SNCA* influence the gene's mRNA levels [13, 23–25].

None of the past *SNCA *expression studies contrasting PD cases and controls used more than 32 total brain samples per brain region (Table 1). Small sample sizes reduce power to detect consistent effects and may have contributed to the conflicting results. We present the largest study to date contrasting *SNCA* expression between PD and control brain samples, with analysis performed for both full-length *SNCA* transcript (140 residue protein isoform, hereafter referred to as SNCA-FL), and total *SNCA *mRNA in the frontal cortex. Additionally, we analyze the relation of eight SNPs in the 5′ and 3′ regions of *SNCA* to *SNCA* expression levels.

## 2. Materials and Methods

### 2.1. Brain Samples

Brain tissue from the frontal cortex Brodmann area 9 was collected from 118 PD cases and 87 control brains. The brain tissue was obtained from three different brain banks: the Harvard Brain Tissue Resource Center (HBTRC) McLean Hospital, Belmont, Massachusetts, the Human Brain and Spinal Fluid Resource Center (HBSFRC) VA West Los Angeles Healthcare Center, California, and the Sun Health Research Institute (SHRI) Sun City, Arizona.

### 2.2. pH Measurements

The pH of all samples was measured following a previously established protocol [26]. A minimum of two pH measurements were taken for each brain sample and the average value of all readings was used.

### 2.3. Neuropathological Information

Neuropathology reports were available for case and control samples. These reports were used to verify the PD diagnosis in the cases, and to evaluate the presence of Alzheimer disease (AD) characteristics in all brain samples. The AD variable for each brain was categorized as 0, 1, or 2 and was determined by a grading of plaques and the Braak score [27, 28]. A value of 0 corresponds to brains that had no indication of Alzheimer pathology, a value of 1 corresponds to brains that had suggestive Alzheimer pathology, and a value of 2 corresponds to brains with unequivocal Alzheimer pathology (Supplementary Table  1 available at doi:10.1155/2012/614212).

### 2.4. Quantitative Real-Time Polymerase Chain Reaction

#### 2.4.1. RNA Extraction and cDNA Synthesis

Total RNA from the brain samples was extracted using the TRIzol reagent (Invitrogen, Carlsbad, CA). The obtained RNA was quantified at 260 nm using a NanoDrop spectrophotometer (Thermo Fisher Scientific, Wilmington, DE). One microgram cDNA was synthesized for each brain sample using the iScriptTM cDNA Synthesis Kit (BIO-RAD, Hercules, CA).

#### 2.4.2. Endogenous Control Gene and the Analysis Method

We considered both the Relative Standard Curve and the ΔΔCt methods for the real-time PCR quantification of *SNCA* expression. *QARS* (encoding for Glutaminyl-tRNA synthetase) was selected as a control gene, given its successful use in previous cortex expression studies [29, 30]. Predesigned TaqMan primers for *QARS *(Hs00909458_g1), *SNCA* transcript NM_000345.2 (Hs00240907_m1, e.g., SNCA-FL), and all *SNCA *transcripts (Hs01103383_m1, total SNCA) were obtained from Applied Biosystems (Foster City, CA). Each sample was run in triplicate for each assay on an ABI PRISM 7900HT Sequence Detection system (Applied Biosystems, Foster City, CA). The control gene did not have the same PCR amplification efficiency as the *SNCA* target assays, which is a requirement for valid ΔΔCt calculations (Validation Experiment, ABI qRT-PCR manual). Therefore, the Relative Standard Curve Method was used to assess the expression data. The standard curves for the three assays were created from pooled cDNA from all available samples and were used to transform the Ct values into quantity units. For each sample that passed the QC filtering criteria, the quantity units for the SNCA-FL and total SNCA assays were standardized by division to the *QARS* control assay quantity value.

#### 2.4.3. DNA Extraction

DNA from the brain samples was extracted using QIAGEN's Puregene Core Kit A (QIAGEN, Valencia, CA) according to the manufacturer's protocol.

#### 2.4.4. Genotyping

Eight SNPs around the *SNCA* gene on chromosome 4 (Table 3) were genotyped in the available brain samples using the TaqMan technology implemented on an ABI PRISM 7900HT Sequence Detection system (Applied Biosystems, Foster City, CA). Pairs of SNPs rs356219-rs356229 (*r* ^2^ = 0.51) and rs4106153-rs1504489 (*r* ^2^ = 0.27) were in modest LD, as calculated by using all available brain samples.

### 2.5. Quality Control

#### 2.5.1. RNA and DNA Extraction

Samples with RNA or DNA extraction yields below 5 *μ*g after several attempts were removed from the study.

#### 2.5.2. RT-PCR

Samples were removed from the study if the variation in expression across the triplicate Ct values for any of the four gene expression assays was larger than 2 (Section 2.4). 

#### 2.5.3. Postmortem Information

Samples were excluded if their PMI information was missing.

#### 2.5.4. Neuropathological Information

Samples were removed when controls showed signs of Lewy bodies, or when PD was not confirmed neuropathologically (e.g., absence of Lewy bodies).

#### 2.5.5. Age at Death

The age at death was available for cases and controls, but the range of values differed by disease status. All controls outside of ±5 years of the PD range (Table 2) were excluded from the case-control contrasts.

#### 2.5.6. Genotyping

All samples with missing genotypes for more than 4 SNPs (less than 50% call rate) were removed from the genotype-expression part of the study. All genotyped SNPs had call rates higher than 92.12%.

#### 2.5.7. Statistical Analyses

The statistical analyses were performed using SAS 9.1 for Windows. The base 10 logarithm of the standardized SNCA-FL and total *SNCA* expression values was used for all analyses, to ensure the normal distribution of data required by the statistical tests performed. The distributions of the log⁡_10_ SNCA-FL and log⁡_10_ total *SNCA* expression values were examined within site, sex, and disease status subgroups. Samples were removed from analysis if they had SNCA-FL and total *SNCA* expression values lower than the 1st quartile minus 1.5∗ interquartile range, or greater than the 3rd quartile plus 1.5∗ interquartile range. In the final sample data-set, the SNCA-FL and total *SNCA *log⁡_10_ transformed expression values did not deviate significantly from the normal distribution (Shapiro-Wilk test), except for two of the total *SNCA* subgroups (SHRI/female/Control and HBTRC/male/PD).

### 2.6. Regression Models

#### 2.6.1. Association of SNCA Expression with Disease Status

We considered several covariates when looking at the association between *SNCA* expression and disease status, including sex, PMI, source of the specimen, pH, AD, and age at death. Since the PMI was highly correlated to the brain bank source (Table 2), the use of either PMI or site as a covariate yielded comparable results in our models. We retained PMI in these models. PMI and pH were selected as covariates because they were found to be associated with *SNCA* expression. Sex was retained due to a prior report, showing differences in *SNCA* expression between men and women [31]. Age at death was retained in the final model because it was significantly associated with *SNCA* expression in controls and was a modest confounder of the relationship between disease status and *SNCA* expression. AD was not associated and was not a confounder of *SNCA* expression; therefore, it was not included in the regression analyses. The interaction between PMI and disease status was included to adjust for the observed variation in *SNCA* expression between PD cases and controls at different PMIs.

#### 2.6.2. Genotyping Analysis

Eight SNPs were tested for association with disease status, as well as for association with SNCA-FL and total *SNCA* expression. Each SNP was evaluated in additive and, whenever the rare homozygote was present, recessive models. Disease association models included adjustment for sex and age at death. Expression models were analyzed for the set of all brains, as well as within only PD cases and only controls. The entire sample analysis included adjustment for disease status, sex, pH, age at death, as well as for the interaction between PMI and disease status. In the analyses stratified by disease status only, sex, pH, age at death, and PMI were included in the model.

## 3. Results

Unless otherwise stated, a significance level of *α* = 0.05 and log_10_ expression values (see Section 2.5.7) were used for all tests.

### 3.1. Samples Excluded from the Final Analyses

One control brain and one PD case from HBTRC were excluded from the study due to low DNA extraction yields. One control sample from HBSFRC and seven control samples from SHRI were excluded from further analyses due to low RNA yields. Eleven PD cases (5 from HBTRC, 5 from SHRI, 1 from HBSFRC) and eleven controls (5 from HBTRC, 5 from SHRI, 1 from HBSFRC) were excluded due to inconsistencies among the Ct values in replicates. One control and one PD case from HBSFRC were discarded due to missing PMI information. One HBTRC control showing Lewy bodies at the neuropathological exam, and one HBTRC PD case with very long duration of disease but no Lewy body pathology were removed from analysis. Four brain samples (1 control from HBTRC, 1 PD from HBSFRC, and 2 PD from SHRI) were outliers for both SNCA-FL and total SNCA expression assays and were, therefore, removed. Four PD cases from HBTRC with missing genotypes for at least 6 SNPs were removed from the genotype-expression analysis only. Eighteen controls from HBTRC that were outside the ±  5 years age at death range for the PD group were removed for the expression analyses. The description of the final set of samples used in the study is presented in Table 2.

### 3.2. Correlations and Associations

SNCA-FL and total SNCA expression values were highly correlated (Pearson correlation *r*  value = 0.76, *P* < 0.0001 in the 165 samples).

**Figure 1:**
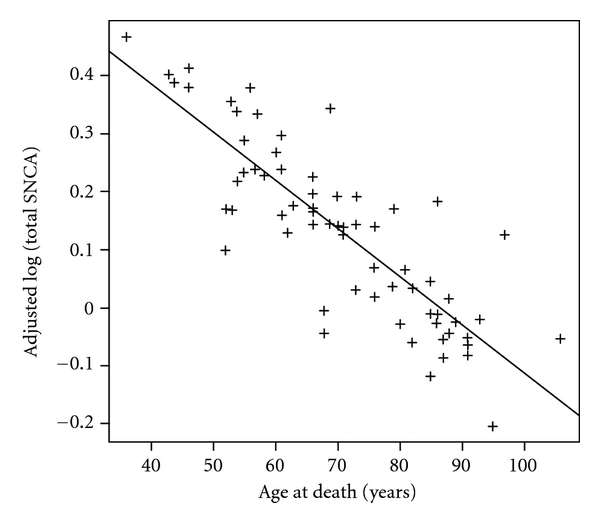
Age at death versus total *SNCA* expression values adjusted for pH, PMI, and sex in 64 controls.

We observed a significant association between age at death and *SNCA* expression values in controls, after adjustment for pH, PMI, and sex (Figure 1 and Supplementary Figure 1; total SNCA: *β* = −0.0059, *P* = 0.0053; SNCA-FL: *β* = −0.0031, *P* = 0.0324). No significant association between age at death and expression values was observed in PD cases after adjusting for pH, PMI, and sex (total SNCA: *P* = 0.84; SNCA-FL: *P* = 0.72). The significant association between *SNCA* expression and age at death remained in controls even after removing samples outside the PD age at death range ±  5 years (total SNCA: *β* = −0.0107, *P* = 0.0075; SNCA-FL: *β* = −0.0056, *P* = 0.0317). Our results confirm the previous finding by Tan et al. (2005), who observed a similar relationship of total *SNCA* expression with age at death in lymphocyte samples from 80 ethnic Chinese control subjects [10].

In a PMI, age at death, and sex-adjusted model, pH was significantly, positively associated with expression of total SNCA in PD cases (*P* < 0.0001) and controls (*P* = 0.002) and with SNCA-FL in PD cases (*P* = 0.0024), but not in controls (*P* = 0.14). We also observed a significant negative association between PMI and both SNCA-FL and total SNCA expression values in PD samples after adjustment for sex, age at death, and pH (*P* < 0.0001 for both SNCA-FL and total SNCA), but not in controls (total SNCA: *P* = 0.96; SNCA-FL: *P* = 0.54).

The correlation between duration of disease in PD samples and *SNCA *expression was not significant (total *SNCA*: *P* = 0.97; SNCA-FL: *P* = 0.64). Additionally, no significant association between the duration of disease in PD samples and *SNCA *expression was observed after adjustment for sex, age at death, and pH.

### 3.3. Expression Results

**Figure 2:**
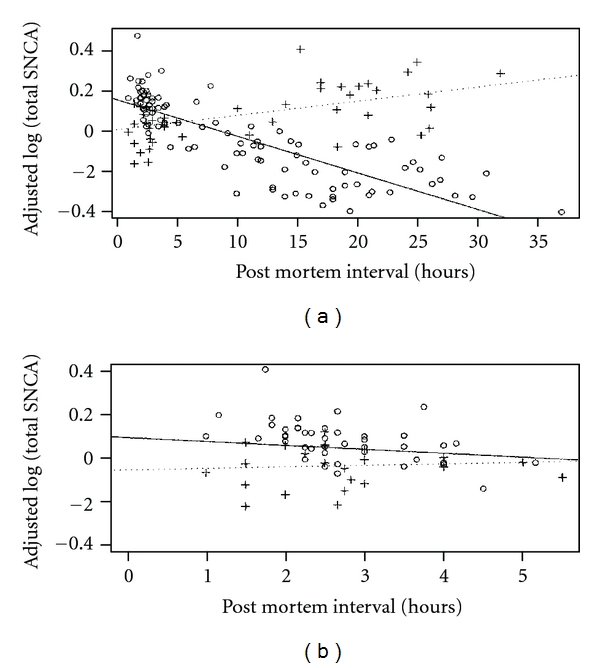
Adjusted total *SNCA* expression values by PMI for (a) all samples and (b) only samples with PMI less or equal to 5.5 hours. Controls are depicted as crosses (dotted regression line) and PD samples as circles (solid regression line).

Interestingly, the PMI was determined to modify the relationship between expression and disease status for both *SNCA*-FL and total *SNCA*. For PMI of 5.5 hours or less (23 controls, 45 cases), PD cases had higher total *SNCA* expression (*β* = 0.1501, *P* = 0.0319) and higher *SNCA* -FL expression (*β* = 0.1195, *P* = 0.0051) than controls. For PMI of 10 hours or more (23 controls, 48 cases), PD cases had lower total *SNCA* expression (*β* = −0.2716, *P* = 0.0005) and lower SNCA-FL expression (*β* = −0.1708, *P* = 0.0093) than controls. The presented results were adjusted for sex, age at death, pH, and PMI. The predicted regression lines for total *SNCA* and SNCA-FL for PD cases and controls after adjustment for age at death, pH, PMI, sex, and disease status-PMI interaction are shown in Figure 2 and Supplementary Figure  2, respectively.

### 3.4. eSNP Results

None of the eight SNPs had a significant nominal *P*-value for association with disease status in our data. Additionally, none of the SNPs had a *P*-value associated with expression that was significant after Bonferroni adjustment for multiple testing. Nevertheless, significant nominal *P*-values (Table 3) were obtained for the following SNPs: rs924033 for SNCA-FL expression using the additive model in controls only, and rs1560488 for total SNCA expression using the recessive model in controls only.

## 4. Discussion

The presence of the *α*-synuclein protein (*α*-syn) in Lewy bodies [34], together with the findings of* SNCA *gene mutations [1–3], *SNCA* gene duplications, and triplications in familial PD [4–6], and *SNCA* SNP associations in PD genome-wide association studies [32] make this gene a focal point of PD research. The association of increased gene dosage with PD risk strongly suggests that increased levels of *α*-syn increase risk for PD. Yet, *SNCA* gene expression studies have yielded inconsistent results with several reporting reduced *SNCA* mRNA levels in PD versus control brains. In this study of 101 PD and 64 control brains, we found significant differences for the effect of post mortem interval on *SNCA *levels in PD. These results may shed light on the previous contradictory expression findings and support the hypothesis that PD is associated with increased *SNCA *levels.

We detected increased expression of the full-length *SNCA *transcript, as well as overall *SNCA* gene expression, in PD compared to control brains at PMI up to 5.5 hours. Additionally, we observed significantly decreased levels of full-length and total *SNCA *in PD compared to control brains at PMI longer than 10 hours. The result obtained for short PMI suggests the presence of biologically increased levels of *SNCA* expression in PD compared to normal brains, while the apparently conflicting findings between short and long PMI groups could be attributable to a more rapid degradation rate of *SNCA, *and possibly other transcripts, in PD brains. The presence of increased RNA degradation activity in PD compared to normal brains is conceivable, given the large differences in expression profiles between normal and affected brains [29]. Additionally, although little information is available on the differential mRNA degradation levels between neurologically healthy and diseased brains, there exists a prior report of correlation between PMI and pH (and indirectly between PMI and certain mRNAs level [26]) in Alzheimer disease, but not in control brains [35].

Previous *SNCA* expression studies have consistently used relatively small numbers of brain samples with mixed PMI values (commonly above 10 hours), precluding an accurate assessment of the effect of PMI [36]. Therefore, the previous conflicting results (Table 1) might be an artifact of both small sample sizes and heterogeneous PMI values.

It is important to note, however, that the different sources of tissue in our study were also related to different PMI values (Table 2). Nearly all of the short PMI samples were from the SHRI brain bank. Therefore, we cannot exclude the possibility that the source of the tissue also influences *SNCA *expression levels. Nevertheless, our statistical analyses included all major variables that are commonly considered in expression studies, and we do not know of any other differences that may exist among the different brain tissue sources and would influence *SNCA* expression. Additionally, given the previous knowledge of frontal cortex homogeneity in terms of expression [11], it is unlikely that variation within the Brodmann area 9 from different brain banks is a factor in the observed findings.

We acknowledge as a possible limitation for our *SNCA *RT-PCR expression study the use of a single control gene. To address this potential problem, we tried to evaluate the obtained RT-PCR expression data by using expression results from a recent microarray study [37]. The microarray experiment was performed on the One-Color Agilent 60-mer Whole Human Genome Microarray, which contains a single 3′ UTR probe for the *SNCA *gene repeated 10 times on the chip. The microarray experiment included a subset of 26 PD and 23 control samples from the RT-PCR study. The range of PMI values for the microarray samples did not allow the interaction study presented in this paper to be tested. Nevertheless, we could evaluate the correlation between the two RT-PCR *SNCA *probes and the median expression value of the microarray *SNCA *probe, which measures total expression of the gene. As expected, the correlation between the total SNCA and the microarray *SNCA* probe (*r* = 0.68, *P* = 6.6*E*−8) was strong and better than the correlation between the SNCA-FL and the microarray *SNCA *probe (*r* = 0.43, *P* = 0.001). These correlation results imply the validity of the RT-PCR data.

Our study suggests that sporadic PD is associated with increased *SNCA* mRNA levels in samples with short PMI. The observation of higher *SNCA* expression in controls among samples with longer PMI suggests that *SNCA* transcripts may degrade more rapidly in PD than in normal brain; this result points to the importance of brain samples with short PMI for an accurate evaluation of RNA levels in PD. Therefore, brain banks such as the Sun Health Research Institute, which can provide samples with very low PMI to the research community, are valuable for future neurodegenerative research.

## Supplementary Material

The Supplementary Materials contain the following information: 1) a description of the neuropathological criteria used to define the Alzheimer disease variable discussed in Section 2.3 of Materials and Methods (Table 1), 2) a scatter plot of age at death and SNCA-FL expression values (after adjustment for pH, PMI, and sex) in controls, which shows a significant association (Figure 1), and 3) the scatter plots and predicted regression lines for SNCA-FL expression (after adjustment for age at death, pH, PMI, sex and disease status-PMI interaction) in PD cases and controls for the whole range of available postmortem intervals and for postmortem intervals up to 5.5 hours (Figure 2).

## Figures and Tables

**Table 1:** Previous brain *SNCA* expression studies.

Study	Method	Brain Region	#Samples (PD/C)	PMI, hours (range)^1^	Expression in PD compared to controls	*SNCA *Transcript
Neystat et al. (1999) [7]	Ribonuclease protection assay	*Substantia nigra*	15 (9/6)	11.55 (4–18) 10.62 (3.5–17)	Decreased, both transcripts	NM_000345.2 NM_007308.1
		Frontal cortex	15 (9/6)	11.55 (4–18) 10.62 (3.5–17)	No significant difference	
Kingsbury et al. (2004) [9]	Semiquantitative *in situ *	*Substantia nigra*	11 (7/4)	23.2 (9.3–56) 33.0 (22–53)	Decreased	NM_000345.2
	Hybridization	Frontal cortex	12 (8/4)	24.4 (10.6–40) 33.0 (22–53)	Decreased	
		Temporal cortex	12 (8/4)	24.4 (10.6–40) 33.0 (22–53)	No significant difference	
Chiba-Falek et al. (2006) [11]	Real-time PCR	Mid-brain	14 (7/7)	16.93 (2.00–22.08) 18.62 (14–24)	Increased	NM_000345.2
		Frontal cortex	7 (4/3)	14 (2–20) 24.66 (22–28)	No significant difference	
Fuchs et al. (2008) [13]	Real-time PCR	*Substantia nigra*	22 (8/14)	All: 25 (N/A)	No significant difference	NM_000345.2
		Cingulate gyrus	32 (13/19)	All: 22 (N/A)	No significant difference	
		Cerebellum	10 (5/5)	All: 15 (N/A)	Decreased	
Beyer et al. (2011) [14]	Real-time PCR	Caudate nucleus	21 (7/14)	6.03 (3.5–7.0) 7.40 (3.5–13.0)	No significant difference	NM_007308.1
		Pons	21 (7/14)	6.03 (3.5–7.0) 7.40 (3.5–13.0)	No significant difference	
		Temporal cortex	21 (7/14)	6.03 (3.5–7.0) 7.40 (3.5–13.0)	No significant difference	

C: Control; PD: Parkinson disease.

NM_000345.2 =140 amino acid isoform; NM_007308.1 =112 amino acid isoform.

^1^The postmortem interval mean and range data for PD samples are on the first line and those for control samples are on the second line. The Fuchs et al. study (2008) only had aggregate mean postmortem interval data available.

**Table 2:** Characteristics of the PD cases and controls included in analysis.

Site	Type	Gender	Age, years (range)	PMI, hours (range)	Tissue pH (range)	PD Duration, years (range)
HBSFRC	2 C	2M	86.5 (80–93)	19.5 (13–26)	6.41 (6.26–6.55)	N/A
	17 PD	9F/8M	82.2 (63–95)	16.3 (9–37)	6.30 (6.02–6.62)	11.5 (4–28)
BTRC	39 C	39M	61.4 (36–106)	21.9 (10–39.6)	6.71 (5.95–7.32)	N/A
	35 PD	35M	76.3 (64–95)	17.9 (6.6–30.7)	6.50 (5.86–7.13)	11.2 (3–23)
SHRI	23 C	13F/10M	84.3 (63–97)	2.68 (1–5.5)	6.71 (6.29–7.13)	N/A
	49 PD	11F/38M	78.5 (64–90)	3.11 (1–10)	6.59 (6.17–7.44)	10.4 (0–40)
All	64 C	13F/51M	70.5 (36–106)	14.9 (1–39.6)	6.70 (5.95–7.32)	N/A
	101 PD	20F/81M	78.3 (63–95)	10.4 (1–37)	6.51 (5.86–7.44)	10.9 (0–40)
Final C Set*	46 C	13F/33M	77.2 (58–97)	11.7 (1–39.6)	6.67 (5.95–7.32)	N/A

C: Control; PD: Parkinson disease.

HBSFRC = Human Brain and Spinal Fluid Resource Center VA West Los Angeles Healthcare Center.

HBTRC = Harvard Brain Tissue Resource Center, McLean Hospital, Belmont, Massachusetts.

SHRI = Sun Health Research Institute in Sun City, Arizona.

*after removing controls with age at death ± 5 years beyond age at death of cases (< age 58 or > age 100).

**Table 3:** Description of the genotyped SNPs and results for association with *SNCA* expression.

SNP	Position (Genome Build 36.3)	Gene	Familial PD GWAS *P*-values [32]	*SNCA* expression estimate	*SNCA* expression min *P*-value	Sample^a^/ Transcript^b^/Model^c^	MAF in expression samples	A1/A2
rs1560488	90,444,858	*GPRIN3*	0.12	0.235	**0.048**	C/T/rec	0.229	T/C
rs4106153	90,463,499	intergenic	9.18 × 10^−5^	−0.048	0.206	All/T/add	0.196	C/A
rs1504489	90,477,611	intergenic	8.42 × 10^−5^	−0.124	0.089	PD/T/rec	0.425	T/G
rs924033	90,654,576	intergenic	0.02	0.165	**0.041**	C/FL/add	0.067	G/T
rs356229	90,825,620	intergenic	5.48 × 10^−5^	−0.099	0.247	C/FL/rec	0.360	C/T
rs356219	90,856,624	intergenic	*2.24 × 10^−6^	0.053 0.040	0.062 0.085	PD/FL/add All/FL/add	0.391	G/A
rs356188	90,910,560	*SNCA*	8.41 × 10^−5^	0.062	0.063	C/FL/add	0.278	C/T
rs3775478	91,061,863	*MMRN1*	6.07 × 10^−5^	0.035 0.017	0.672 0.672	C/T/add All/FL/add	0.090	G/A

*imputed SNP result for published *SNCA* eSNP [13, 33].

^a^PD-Case, C-Control, All-Combined sample.

^b^FL-full length or T-total.

^c^additive or recessive SNP model.

A1-minor allele, A2–major allele.
