# Supplementary material for: Postmortem Interval Influences α-Synuclein Expression in Parkinson Disease Brain
Source: Parkinsons Dis. 2012 Mar 13;2012:614212. doi: 10.1155/2012/614212 (PMC3317023; doi:10.1155/2012/614212)
Supplement: Supplementary file 1 — The Supplementary Materials contain the following information: 1) a description of the neuropathological criteria used to define the Alzheimer disease variable discussed in Section 2.3 of Materials and Methods (Table 1), 2) a scatter plot of age at death and SNCA-FL expression values (after adjustment for pH, PMI, and sex) in controls, which shows a significant association (Figure 1), and 3) the scatter plots and predicted regression lines for SNCA-FL expression (after adjustment for age at death, pH, PMI, sex and disease status-PMI interaction) in PD cases and controls for the whole range of available postmortem intervals and for postmortem intervals up to 5.5 hours (Figure 2). [file 614212.f1.pdf]

Post-mortem Interval Influences α-Synuclein Expression in Parkinson Disease Brain

Supplementary Material

Supplementary Table 1: *Encoding of AD variable for the final sample using neuropathological information*

AD Value	LA	McLean	Sun Health
**2**	Information in the Neuropathological Diagnosis and in the Microscopic Examination sections	Abundant plaques in the hippocampus region<br>Age and Braak stage considered	Plaque Total ≥ 4.5<br>Braak ≥ 4<br>or<br>Plaque Total ≥ 10<br>Braak = 3
	1 PD, 0 controls	3 PD, 0 controls	6 PD*, 0 controls
**1**	Information in the Neuropathological Diagnosis and in the Microscopic Examination sections	Moderate plaques in the hippocampus region<br>Age and Braak stage considered	Plaque Total ≥ 4.5<br>Braak = 3<br>or<br>Plaque Total ≥ 8<br>Braak = 2
	2 PD, 1 control	13 PD, 5 controls	9 PD, 0 controls
**0**	No evidence for plaques		
	14 PD, 1 control	19 PD, 34 controls	33 PD, 23 controls

**all 6 brains had B Cerad Neuritic Plaque scores assigned to them*

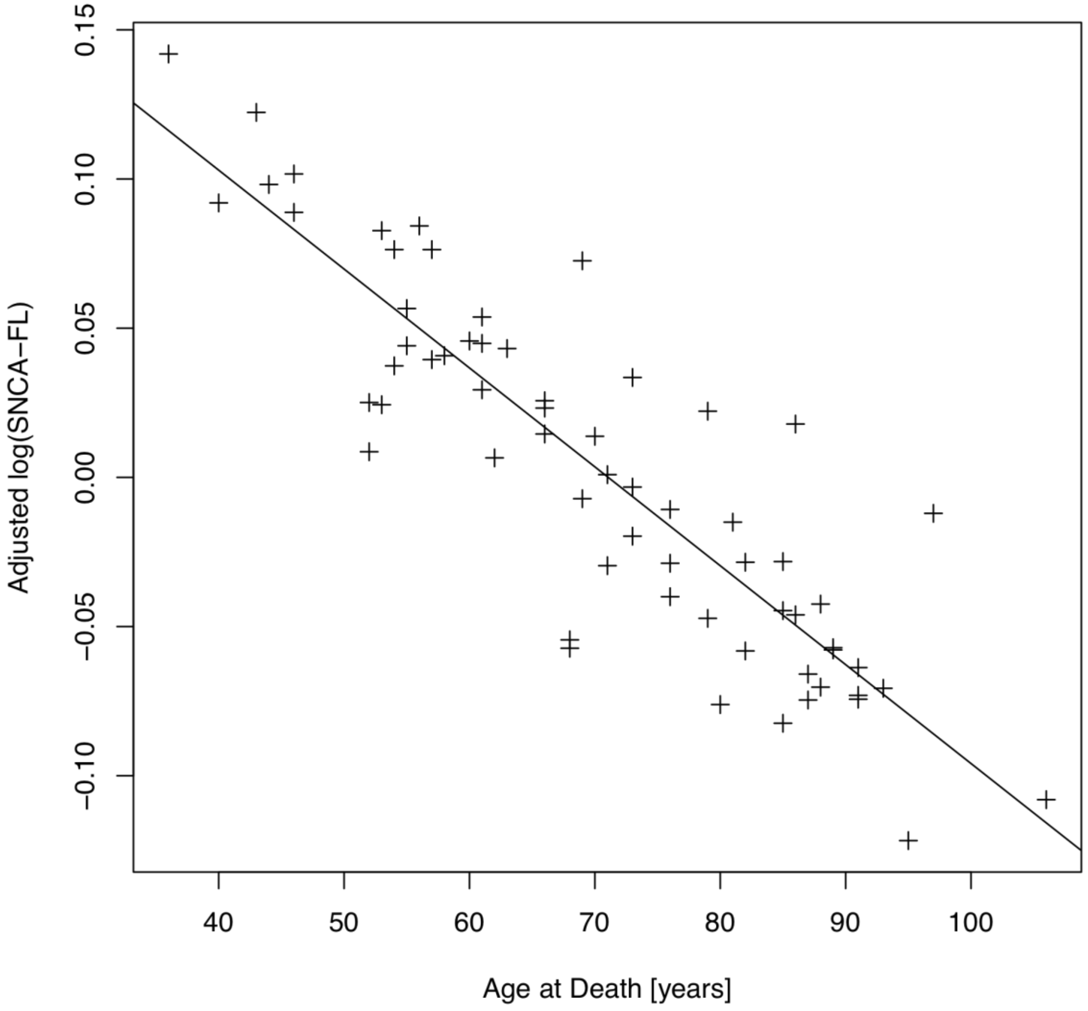

Supplementary Figure 1: *Age at death versus SNCA-FL expression values adjusted for pH, PMI, and sex in 64 controls.*

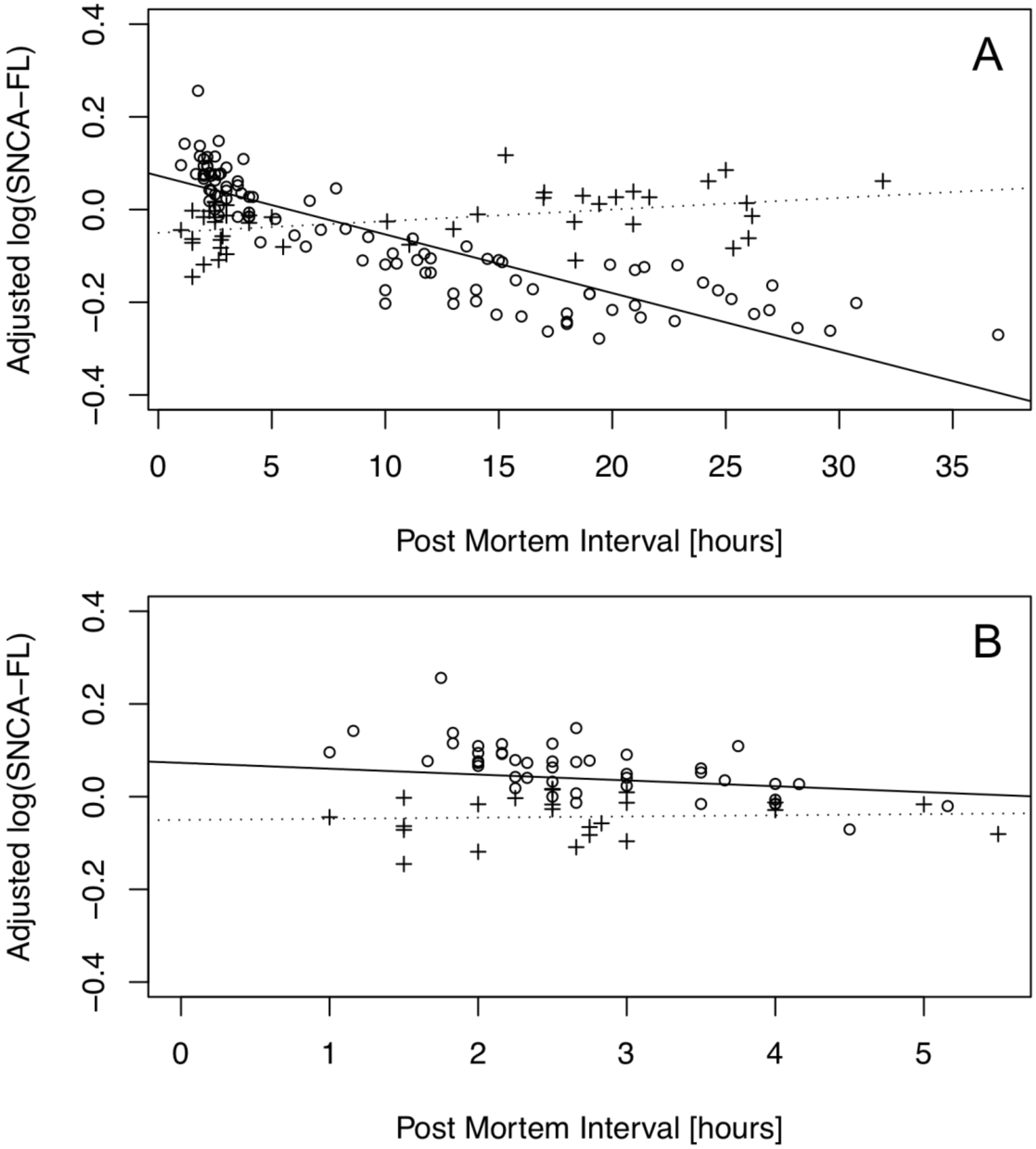

Supplementary Figure 2: *Adjusted SNCA-FL expression values by PMI for A) all samples and B) only samples with PMI less or equal to 5.5 hours. Controls are depicted as crosses (dotted regression line) and PD samples as circles (solid regression line).*
